# Healthcare Cost Savings Estimator Tool for Chronic Disease Self-Management Program: A New Tool for Program Administrators and Decision Makers

**DOI:** 10.3389/fpubh.2015.00042

**Published:** 2015-04-27

**Authors:** SangNam Ahn, Matthew Lee Smith, Mary Altpeter, Lindsey Post, Marcia G. Ory

**Affiliations:** ^1^Division of Health Systems Management and Policy, School of Public Health, The University of Memphis, Memphis, TN, USA; ^2^Department of Health Promotion and Community Health Sciences, Texas A&M University Health Science Center, School of Public Health, College Station, TX, USA; ^3^Department of Health Promotion and Behavior, College of Public Health, The University of Georgia, Athens, GA, USA; ^4^Center for Health Promotion and Disease Prevention, University of North Carolina at Chapel Hill, Chapel Hill, NC, USA

**Keywords:** chronic disease, chronic disease self-management program, healthcare cost, healthcare cost savings estimator tool, return on investment

## Abstract

Chronic disease self-management education (CDSME) programs have been delivered to more than 100,000 older Americans with chronic conditions. As one of the Stanford suite of evidence-based CDSME programs, the chronic disease self-management program (CDSMP) has been disseminated in diverse populations and settings. The objective of this paper is to introduce a practical, universally applicable tool to assist program administrators and decision makers plan implementation efforts and make the case for continued program delivery. This tool was developed utilizing data from a recent *National Study of CDSMP* to estimate national savings associated with program participation. Potential annual healthcare savings per CDSMP participant were calculated based on averted emergency room visits and hospitalizations. While national data can be utilized to estimate cost savings, the tool has built-in features allowing users to tailor calculations based on their site-specific data. Building upon the *National Study of CDSMP’s* documented potential savings of $3.3 billion in healthcare costs by reaching 5% of adults with one or more chronic conditions, two heuristic case examples were also explored based on different population projections. The case examples show how a small county and large metropolitan city were not only able to estimate healthcare savings ($38,803 for the small county; $732,290 for the large metropolitan city) for their existing participant populations but also to project significant healthcare savings if they plan to reach higher proportions of middle-aged and older adults. Having a tool to demonstrate the monetary value of CDSMP can contribute to the ongoing dissemination and sustainability of such community-based interventions. Next steps will be creating a user-friendly, internet-based version of *Healthcare Cost Savings Estimator Tool: CDSMP*, followed by broadening the tool to consider cost savings for other evidence-based programs.

## Background and Rationale

Adults with chronic conditions are the primary users of healthcare in US and account for two-thirds of total healthcare spending (1). Healthcare costs in US, as measured by the percentage of gross domestic product (GDP), essentially doubled in <30 years from 9.2% in 1980 to 17.6% in 2009 (2). People with three or more chronic conditions have 14.6 times more hospital stays than patients with no chronic conditions, and patients with co-morbidities spend 25 times more nights in the hospital than adults with no chronic conditions (3). Coupled with our rapidly aging society, this trend, if not curtailed, will lead to one of every three dollars spent in America paying for healthcare by 2040, with at least 65% of that spending going toward patients with multiple chronic conditions (4).

The chronic disease self-management program (CDSMP) has been introduced to help patients with chronic conditions improve their health behaviors, enhance their health outcomes, and reduce healthcare utilization (5, 6). Topics covered in CDSMP include coping skills and symptom control (7). Coping strategies such as action planning and feedback, behavior modeling, problem-solving techniques, and decision-making are applicable to all chronic conditions. CDSMP participants are also taught how to control their symptoms through relaxation techniques, healthy eating, sleep and fatigue monitoring, medication management, exercise, and improved communication with providers. Led by two peer facilitators, CDSMP is a highly interactive program that engages participants for six weekly sessions for two and a half hours per session (8). Each CDSMP delivery site recruited people for workshops in their usual manner including self-referrals from flyers, brochures, and health fairs as well as referrals from organizations serving older adults (e.g., senior centers, social service organizations) (8). Additional information regarding sampling, recruitment, training, and fidelity assessment can be found in previous work done by the authors (8).

Since its introduction, CDSMP has been made available in all US states (9) and 25 countries (10–12). More recent studies documented CDSMP participants’ improvements in the Triple Aim components of healthcare reform (i.e., better healthcare, better health outcome, better value) (8). CDSMP can improve healthcare delivery and patient experience through increased patient–physician communication, better education about medication utilization, and patient empowerment, and self-efficacy (8, 13).

There is also a rapidly growing body of evidence demonstrating substantially better health outcomes for CDSMP participants, which include improved self-reported general health, fewer social activity limitations, more physical activity, and decreases in depression, fatigue, and pain (8, 13, 14). These benefits have been demonstrated among participants with a variety of chronic conditions, across the full spectrum of socioeconomic status, and in multiple types of delivery settings (14). To better value, healthcare-related cost savings are achieved as healthcare utilization decreases, evidenced by reduced hospitalizations, emergency room (ER) visits, and lengths of hospital stays (14, 15). A recent study estimated annual net cost savings of $364 per CDSMP participant, which would amount to a national savings of $3.3 billion assuming 5% of adults with one or more chronic conditions participated in the intervention (15).

While the cost savings associated with CDSMP delivery and participation can be calculated, there is no universal tool or standardized method for easily estimating program cost savings among CDSMP participants. Such a tool would be of great benefit to program administrators responsible for allocating resources for evidence-based programs. More specifically, a tool estimating cost savings including training, personnel, and material costs (16) could help program deliverers estimate the average per participant program costs. Even more, if there were a tool that guided users step-by-step through the process and allowed them to tailor estimates by “filling in the blanks” based on their specifications and available data, program administrators could more confidently demonstrate the effectiveness of CDSMP at containing costs in their communities and service areas. Additionally, the tool could help program administrators be strategic when selecting participant recruitment goals and/or targeting particular participant groups (e.g., based on their healthcare utilization patterns or geographic location), identify returns on investment, justify funding requests, and prepare for program scalability and sustainability within their organization and/or community. To support these strategic planning efforts, this paper: (1) describes the development of the *Healthcare Cost Savings Estimator Tool: CDSMP* (i.e., tool); (2) illustrates how the tool can be tailored by users and introduces two heuristic case examples to show how context impacts potential cost savings; and (3) describes the recommended uses of the tool and potential challenges to be considered.

## Methods: Healthcare Cost Savings Estimator Tool Origins and Creation

Data from the *National Study of CDSMP*, conducted from 2010 to 2012 among 22 licensed sites within 17 states, were used to create the tool. The *National Study of CDSMP* was part of the American Recovery and Reinvestment Act of 2009 (i.e., ARRA) *Communities Putting Prevention to Work: Chronic Disease Self-Management Program initiative* (17). Data from 1,170 CDSMP participants were used to estimate health cost savings associated with the program (15). Of the 1,170 participants at baseline, approximately 77% (*n* = 903) and 71% (*n* = 825) completed the 6- and 12-month assessments, respectively (8). On each assessment, participants were asked to self-report any ER visits and hospitalizations in the previous 6 months. These items were included to identify changes in participants’ healthcare utilization at three time points. The health benefits and financial effects of this *National Study* have been documented in previous studies (8, 15).

Based on data from the *National Study of CDSMP*, a six-step process was developed for assessing potential cost savings (15). An excel-based tool was constructed that used *National Study* data to summarize potential national savings as a default; however, users are able to override the default by inputting their own numbers to estimate the savings accrued by offering CDSMP in their service area. More details about the data required of users for tailored estimates are provided below. This excel-based tool is publicly available at: http://cdsmp-cost-tool.herokuapp.com/static/files/CDSMP_Cost_Estimator.xls.

## Healthcare Cost Savings Estimator Tool

### Generic six-step healthcare cost savings estimator tool modeled from the national study of CDSMP

The following six-step method was developed as a practical way for identifying program costs and potential cost savings for evidence-based programs, utilizing the *National Study of CDSMP* as the case example (15). These data are the basis of the tool’s creation.

Step 1: examine the pattern of ER visits and hospitalizations among CDSMP participants (*n* = 1,170) in the first and second 6-month periods.
  Table 1 shows changes in ER visits from baseline (18%) to 6 months (13%) to 12 months (13%). ER visits between baseline to 6 months (5%) and baseline to 12 months (5%) were significantly reduced (15). Table 1 also shows changes in hospitalizations from baseline (14%) to 6 months (11%) to 12 months (14%). Hospitalizations between baseline and 6 months (3%) were significantly reduced (15).
Step 2: Identify age-adjusted mean costs for ER visits and hospitalizations from 2010 Medical Expenditure Panel Survey (MEPS).
  Table 1 shows mean costs for ER visits and hospitalizations from 2010 MEPS. The MEPS data were selected for this study because this is the most complete source of data related to the cost and use of healthcare in US at the time of this study (18). First, we identified the age distribution in the *National Study of CDSMP*: 10% were 18-44 years of age, 31% were 45-64 years of age, and 59% were 65 years of age or older. Then we identified mean costs of ER visits by the aforementioned age categories from the 2010 MEPS dataset and found $1,513 as the age-adjusted cost of ER visits. The age-adjusted value was used to calculate a more accurate cost of ER visits based on the age distribution and mean costs of ER visits of each age category. Thus, total cost savings associated with ER visits per person at two time periods amounted to $151.31 [first 6 months (5% reduction × $1,513) + second 6 months (5% reduction × $1,513)]. Using the same MEPS dataset, we identified $18,750 as an age-adjusted cost of hospitalizations, and total cost savings associated with hospitalizations per person at two time periods amounted to $562.50 [first 6 months (3% reduction × $18,750) + second 6 months (0% × $18,750)].
Step 3: estimate costs saved from reduced ER visits and hospitalizations for two 6-month periods of CDSMP.
  Table 1 shows that $714 was the potential annual healthcare savings per CDSMP participant from averting ER visits ($151) and hospitalizations ($563).
Step 4: Estimate average annual CDSMP costs.
  Table 1 shows that we suggest $350 as the estimated program delivery cost per person in the *National Study of CDSMP* based on best estimates from experts and field reports (15). It should be noted that estimates were based on the cost of $3,500 per CDSMP workshop, thus the cost per participant ranged from $219 for workshops of 16 participants, $350 for workshops of 10 participants, and $583 for workshops of 6 participants.
Step 5: Deduct the average annual CDSMP costs (#4) from the estimated cost savings due to reduced ER visits and hospitalizations (#3).
  The potential annual net healthcare cost savings of $364 per participant was found by deducting the annual per participant program costs ($350) from the estimated annual per participant healthcare savings ($714) (Table 1).
Step 6: Extrapolate to national savings using Census data among American adults (with a population size of 234.5 million age 18 years and above) having at least 1 chronic condition combined with MEPS data.

**Table 1:** **Healthcare cost savings estimator tool: the national study of CDSMP**.

CDSMP health cost savings estimator^a^			

National study case example			

	*N*	%	Change in %
**1. EXAMINE THE PATTERN OF HEALTH CARE UTILIZATION WITHIN YOUR POPULATION**			
**Emergency room (ER) visits**			
Include number of participants at baseline	1170		
Include number of participants reported visiting ER at baseline	211	18%	
Include number of participants at 6 months	903		
Include number of participants reported visiting ER at the first 6 months	118	13%	5%
Include number of participants at 12 months	825		
Include number of participants reported visiting ER at the second 6 months	108	13%	5%
**Hospitalizations**			
Include number of participants reporting hospitalization at baseline	164	14%	
Include number of participants reporting hospitalization at the first 6 months	100	11%	3%
Include number of participants reporting hospitalization at the second 6 months	116	14%	0%
**2. IDENTIFY MEAN COSTS FOR HEALTH CARE UTILIZATION FROM 2010 MEDICAL EXPENDITURE PANEL SURVEY (MEPS)**			
**Age distribution**			
Include % for those 18–44 years of age	10%		
Include % for those 45–64 years of age	31%		
Include % for those 65+ years of age	59%		
**ER visits**			
Mean costs of ER visits for those 18–44 years of age	$ 1,465.00		
Mean costs of ER visits for those 45–64 years of age	$ 1,738.00		
Mean costs of ER visits for those 65+ years of age	$ 1,403.00		
Age-adjusted cost of ER visits	$ 1,513.05		
Cost savings associated with ER visits per person at the first 6 months	$ 75.65		
Cost savings associated with ER visits per person at the second 6 months	$ 75.65		
Total cost savings associated with ER visits per person at two time periods	$ 151.31		
**Hospitalizations**			
Mean costs of hospitalizations for those 18–44 years of age	$ 11,501.00		
Mean costs of hospitalizations for those 45–64 years of age	$ 21,462.00		
Mean costs of hospitalizations for those 65+ years of age	$ 18,554.00		
Age-adjusted cost of hospitalizations	$ 18,750.18		
Cost savings associated with hospitalizations per person at the first 6 months	$ 562.51		
Cost savings associated with hospitalizations per person at the second 6 months	$–		
Total cost savings associated with hospitalizations per person at two time periods	$ 562.51		
**3. ESTIMATE COSTS SAVED FROM REDUCED UTILIZATION FOR THE PERIOD OF TIME YOU ARE INTERESTED IN EXAMINING**			
Based on national information, potential annual health care savings per CDSMP participant from averting ER visits ($ 151.31) and hospitalizations ($ 562.51) can be estimated	$ 713.81		
**4. ESTIMATE AVERAGE ANNUAL PROGRAM DELIVERY COSTS**			
Estimated program delivery costs per person in the National CDSMP study	$ 350.00		
**5. DEDUCT ANNUAL PROGRAM COSTS FROM ESTIMATED HEALTH CARE UTILIZATION SAVINGS**			
Based on national information and using average CDSMP costs per participant ($ 350.00), net cost savings related to ER visits and hospitalizations per CDSMP participant can be estimated	$ 363.81		
**6. EXTRAPOLATE TO NATIONAL SAVINGS USING CENSUS DATA COMBINED WITH MEPS DATA**			
Number of American adults from census data by age	234,564,071	100%	
18–44	112,806,642	48%	
45–64	81,489,445	35%	
65+	40,267,984	17%	
Estimated % of American adults having at least 1 chronic condition from MEPS data by age	77%		
18–44	71%		
45–64	84%		
65+	94%		
Number of American adults aged 18 and older having at least one chronic condition	180,614,335		
Cost savings if you could reach ALL American adults age 18+ having at least one chronic condition	$ 65,709,373,342.03		
Include % of this population you want to reach	5 %		
Based on per participant program annual net savings ($ 363.81) for the population you want to reach (5%), national health care savings can be estimated	$ 3,285,468,667.10		

*^a^Be aware of potential limitations when presenting your data*.

*The cost estimates presented must be treated as general estimates, as they are not based on precise cost expenditures. Yet, we feel they are robust for purposes of providing ballpark health care utilization costs, program delivery expenses, and estimated net savings to support the widespread dissemination and sustainability of evidence-based chronic disease self-management programs*.

Table 1 shows the amount of money that might be saved by implementing the program nationally. To calculate this figure, we extrapolated from per participant annual net savings to national savings using Census and MEPS data. We first identified the age distribution of American adults from 2010 Census data: 18–44 (112.8 million, 48%), 45–64 (81.5 million, 35%), and 65+ (40.3 million, 17%). From the 2010 MEPS data, we tallied percentages of American adults having at least 1 chronic condition: 18–44 (71%), 45–64 (84%), and 65+ (94%). Thus, the age-adjusted number of American adults aged 18 and older having at least 1 chronic condition was 180.6 million [i.e., (112.8 million × 71%) + (81.5 million × 84%) + (40.3 million × 94%)]. Finally, $3.3 billion in healthcare costs may be saved by averting ER visits and hospitalizations if the CDSMP could reach 5% of this population (180.6 million × 5% × $364). It is also important to note that the national extrapolation in Step 6 can be replaced by local projections based on participant reach and age distributions of those projected participants.

## Tailoring the Healthcare Cost Savings Estimator Tool

The information requested of users wanting to tailor their region-specific estimates is described in Table 2. Users are asked to provide data points including the number of CDSMP participants, ER visits, and hospitalizations at baseline, 6- and 12-month; participant age distribution at baseline; and estimated program delivery cost per participant. This information will be used to estimate net cost savings in Step 5 based on their current data sources. In Step 6, this tool can be further tailored by estimating new net cost savings and projecting total healthcare net savings in the next 12 months based on the expected number of participants (e.g., 200) to be enrolled and their anticipated age distribution (e.g., increasing reach of middle-aged participants by 10%).

**Table 2:** **Data points, data sources, formats, and recommendations needed for users to tailor cost estimates**.

Data point	Data source	Format in tool	Recommendation for measurement
Number of CDSMP participants	User’s assessment data	Open-ended	Collected at baseline and 6 months (and 12 months, if possible)
Number of ER visits	User’s assessment data	Open-ended	Collected at baseline and 6 months (and 12 months, if possible)
Number of hospitalizations	User’s assessment data	Open-ended	Collected at baseline and 6 months (and 12 months, if possible)
Participant age distribution	User’s assessment data	Open-ended	Categorized as 18–44, 45–64, and 65 years and older
Estimated program delivery cost (per participant)	User’s administrative data	Drop-down menu	Choices of $219, $250, $292, $350, $438, $583, or open-ended (override)
Number of eligible individuals (aged 18+ years with 1+ chronic conditions) to be served by CDSMP in next 12 months	User’s projection about reach	Open-ended	Open-ended

The data points described above are derived from various data sources including the user’s assessment data (i.e., collected from participants using questionnaires at baseline and follow-up time points); user’s administrative data (i.e., gathered from delivery sites and administrative records about workshop characteristics); and the user’s projections about per participant cost to deliver the program and reach (i.e., the projected number of participants (as well as the new age distribution of participants) the user anticipates enrolling in the forthcoming 12-month period).

The tool comes complete with a set of step-by-step instructions about data to be entered for tailored estimates. Drop-down menus are provided to ensure default values (e.g., those calculated from the *National Study of CDSMP* described above) exist from which to calculate estimates; however, users can override the drop-down menu options by entering their own responses from their data. The more user data that is entered, the more tailored the cost savings estimates will be. It should be noted that cost estimates generated with this Tool are only estimates and the Tool *does not* calculate actual cost savings. It is also noted that there should be at least 100 participants to make the estimates stable.

Two heuristic case examples (i.e., a small county and a metropolitan city) are described below to show how users can utilize the Tool with their own data to create tailored cost savings estimates for their existing and future CDSMP participant populations. These examples reinforce how the context and methods of CDSMP delivery impact potential cost savings. They also demonstrate the value of the Tool for demonstrating potential savings when the age distribution of the projected participant population is adjusted to target older age groups. One case example concerns the Department of Public Health located in a small county while the other case example pertains to an academic institution located in an urban area.

### Case example #1 (department of public health in a small county)

Ms. Jones is the director of the Department of Public Health in a small county with a population size of 7,774, in which 56% of adults are 18–44 years of age, 23% are 45–64 years of age, and 21% are 65 years of age or older according to the Census. She wants to know how much her CDSMP program might be reducing healthcare costs by averting ER visits and hospitalizations among participants (*n* = 125). Ms. Jones also wants to know how much healthcare costs could be saved if she knows the expected number of participants (*n* = 200) to be enrolled in CDSMP in next 12 months and the age distribution of these participants. The six-step process taken by Ms. Jones utilizing the excel-based tool is described below. Ms. Jones entered relevant numbers marked in diagonal stripe based on her data and projections for her target region/service area (Table 3). She consulted her records and the recollection of her colleagues and partners to gather data including number of CDSMP participants (at baseline, 6 and 12 months), ER visits, hospitalizations, baseline age distribution, estimated program delivery cost, and the expected number of participants (and their anticipated age distribution) to be enrolled in CDSMP in next 12 months.

Step 1: Examine the pattern of ER visits and hospitalizations among CDSMP participants (*n* = 125) in the first and second 6-month periods
  Table 3 shows changes in ER visits from baseline (16%) to 6 months (11%) to 12 months (11%). ER visits were reduced between baseline to 6 months (5%) and baseline to 12 months (5%). Table 3 also shows changes in hospitalizations from baseline (12%) to 6 months (10%) to 12 months (11%). Hospitalizations were also reduced between baseline and 6 months (2%) and baseline to 12 months (1%).
Step 2: Identify mean costs for ER visits and hospitalizations from 2010 medical expenditure panel survey (MEPS)
  Table 3 shows mean costs for ER visits and hospitalizations from 2010 MEPS by taking into account the age distribution of the small county as noted above. Then Ms. Jones identified mean costs of ER visits by the aforementioned age categories from the 2010 MEPS dataset and found $1,514.77 as the age-adjusted cost of ER visits. Thus, total cost savings associated with ER visits per person through the two time periods amounted to $151.48 [first 6 months (5% reduction × $1,514.77) + second 6 months (5% reduction × $1,514.77)]. Using the same MEPS dataset, she identified $15,273.16 as an age-adjusted cost of hospitalizations, and total cost savings associated with hospitalizations per person through two time periods amounted to $458.19 [first 6 months (2% reduction × $15,273.16) + second 6 months (1% × $15,273.16)].
Step 3: Estimate costs saved from reduced ER visits and hospitalizations for two 6-month periods of CDSMP
  Table 3 shows that $609.67 was the potential annual healthcare savings per CDSMP participant by averting ER visits ($151.48) and hospitalizations ($458.19).
Step 4: Estimate average annual CDSMP costs
  Table 3 shows that Ms. Jones suggests $438 as the estimated program delivery costs per person based on the average number of participants in each workshop and the organizational capacity of providing CDSMP.
Step 5: Deduct the average annual CDSMP costs (#4) from the estimated cost savings from reduced ER visits and hospitalizations (#3)
  The potential annual net healthcare cost savings of $171.67 per participant was found by deducting the annual per participant program cost ($438) from the estimated annual per participant healthcare savings ($609.67) (Table 3).
Step 6: Project healthcare cost savings based on the expected number of participants to be enrolled in next 12 months and their anticipated age distribution.

**Table 3:** **Healthcare cost savings estimator tool: a small county**.

Healthcare cost savings estimator tool: CDSMP^a^						

National study case example				Your local example: if you have data, please enter relevant numbers in cells marked in diagonal stripe for your population. This spreadsheet will make automatic calculations for you.		

	*N*	%	Change in %	*N*	%	Change in %
**1. EXAMINE THE PATTERN OF HEALTH CARE UTILIZATION WITHIN YOUR POPULATION**						
**Emergency room (ER) visits**						
Include number of participants at baseline	1170			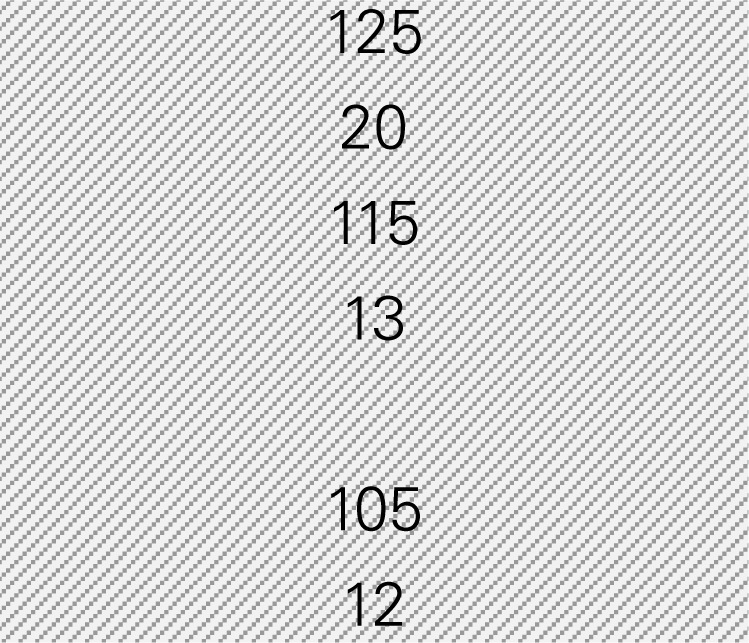		
Include number of participants reported visiting ER at baseline	211	18%			16%	
Include number of participants at 6 months	903					
Include number of participants reported visiting ER at the first 6 months	118	13%	5%		11%	5%
Include number of participants at 12 months	825					
Include number of participants reported visiting ER at the second 6 months	108	13%	5%		11%	5%
**Hospitalizations**						
Include number of participants reporting hospitalization at baseline	164	14%		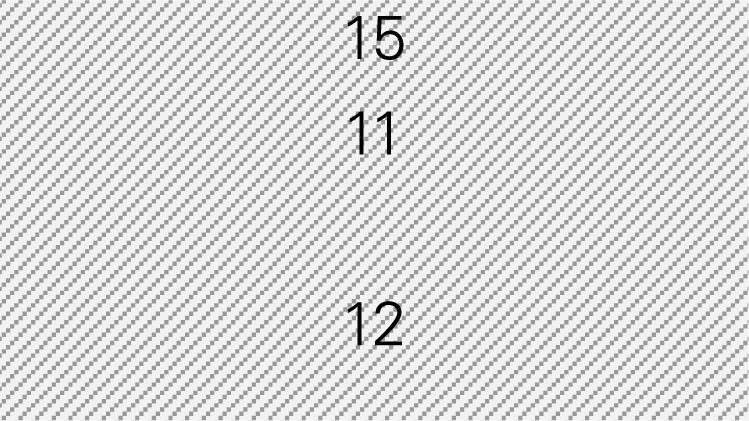	12%	
Include number of participants reporting hospitalization at the first 6 months	100	11%	3%		10%	2%
Include number of participants reporting hospitalization at the second 6 months	116	14%	0%		11%	1%
**2. IDENTIFY MEAN COSTS FOR HEALTH CARE UTILIZATION FROM 2010 MEDICAL EXPENDITURE PANEL SURVEY (MEPS)**						
**Age distribution**				Indicate the age distribution for your population		
Include % for those 18–44 years of age	10%			Indicate % for those 18–44	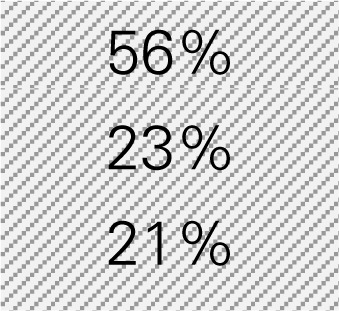	
Include % for those 45–64 years of age	31%			Indicate % for those 45–64		
Include % for those 65+ years of age	59%			Indicate % for those 65+		
**ER visits**			
Mean costs of ER visits for those 18–44 years of age	$ 1,465.00			$ 1,465.00		
Mean costs of ER visits for those 45–64 years of age	$ 1,738.00			$ 1,738.00		
Mean costs of ER visits for those 65+ years of age	$ 1,403.00			$ 1,403.00		
Age-adjusted cost of ER visits	$ 1,513.05			$ 1,514.77		
Cost savings associated with ER visits per person at the first 6 months	$ 75.65			$ 75.74		
Cost savings associated with ER visits per person at the second 6 months	$ 75.65			$ 75.74		
Total cost savings associated with ER visits per person at two time periods	$ 151.31			$ 151.48		
**Hospitalizations**			
Mean costs of hospitalizations for those 18–44 years of age	$ 11,501.00			$ 11,501.00		
Mean costs of hospitalizations for those 45–64 years of age	$ 21,462.00			$ 21,462.00		
Mean costs of hospitalizations for those 65+ years of age	$ 18,554.00			$ 18,554.00		
Age-adjusted cost of hospitalizations	$ 18,750.18			$ 15,273.16		
Cost savings associated with hospitalizations per person at the first 6 months	$ 562.51			$ 305.46		
Cost savings associated with hospitalizations per person at the second 6 months	$–			$ 152.73		
Total cost savings associated with hospitalizations per person at two time periods	$ 562.51			$ 458.19		
**3. ESTIMATE COSTS SAVED FROM REDUCED UTILIZATION FOR THE PERIOD OF TIME YOU ARE INTERESTED IN EXAMINING**						
Based on national information, potential annual health care savings per CDSMP participant from averting ER visits ($ 151.31) and hospitalizations ($ 562.51) can be estimated	$ 713.81			Potential annual health care savings ($ 151.48 + $ 458.19)	$ 609.67	
**4. ESTIMATE AVERAGE ANNUAL PROGRAM DELIVERY COSTS**						
Estimated program delivery costs per person in the National CDSMP study	$ 350.00			Select your closest program cost per person from the drop-down menu	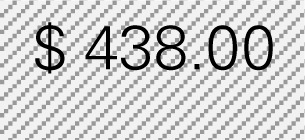	
**5. DEDUCT ANNUAL PROGRAM COSTS FROM ESTIMATED HEALTH CARE UTILIZATION SAVINGS**						
Based on national information and using average CDSMP costs per participant ($ 350.00), net cost savings related to ER visits and hospitalizations per CDSMP participant can be estimated	$ 363.81			Net cost savings ($ 609.67 − $ 438.00)	$ 171.67	
**6. EXTRAPOLATE TO NATIONAL SAVINGS USING CENSUS DATA COMBINED WITH MEPS DATA** **6. CALCULATE YOUR SAVINGS BASED ON POPULATION TO REACH AND NEW AGE DISTRIBUTION**						
Number of American adults from Census data by age	234,564,071	100%		Number of potential participants reflecting their age distribution	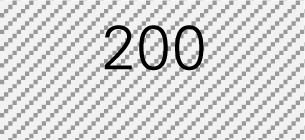	100%
18-44	112,806,642	48%		18–44	102	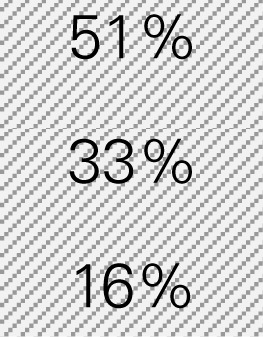
45-64	81,489,445	35%		45–64	66	
65+	40,267,984	17%		65+	32	
Estimated % of American adults having at least 1 chronic condition from MEPS data by age	77%			Net Cost Savings based on population to reach and new age distribution	194.02	
18-44	71%					
45-64	84%					
65+	94%					
Number of American adults aged 18 and older having at least 1 chronic condition	180,614,335					
Cost savings if you could reach ALL American adults age 18+ having at least 1 chronic condition	$ 65,709,373,342.03					
Include % of this population you want to reach	5%					
Based on per participant program annual net savings ($ 363.81) for the population you want to reach (5%), national health care savings can be estimated	$ 3,285,468,667.10			Your healthcare net cost savings by averting ER visits and hospitalizations attributed to CDSMP	$38,803.06	

*^a^Be aware of potential limitations when presenting your data*.

*The cost estimates presented must be treated as general estimates, as they are not based on precise cost expenditures. Yet, we feel they are robust for purposes of providing ballpark health care utilization costs, program delivery expenses, and estimated net savings to support the widespread dissemination and sustainability of evidence-based chronic disease self-management programs*.

After acknowledging $171.67 as per person net cost savings among CDSMP participants in Step 5, Ms. Jones wants to project healthcare cost savings when reaching 200 people in the next 12 months (Table 3). She also decides to recruit 10% more middle-aged adults (i.e., from 23 to 33%) after realizing that the costs of ER visits and hospitalization for this population is more expensive than their younger or older counterparts (also shown in Table 3). As a result, when reaching more middle-aged population [i.e., 33% compared to younger (51%) and older adults (16%)], Ms. Jones estimates $194.02 as the new net cost savings, and concludes CDSMP could potentially help save $38,804 (i.e., 200 × $194.02). This equates to approximately $4,000 more healthcare cost savings than the age distribution of her existing CDSMP participant pool.

### Case example #2 (academic institution in an urban area)

Mr. Smith is a director of the Healthy Aging Network at an academic institution providing CDSMP in a metropolitan city with a population size of 940,764, in which 72% of adults are 18–44 years of age, 16% are 45–64 years of age, and 12% are 65 years of age or older according to the Census. He wants to know how much his CDSMP program could potentially reduce healthcare costs by averting ER visits and hospitalizations among participants (*n* = 500). Mr. Smith also wants to project healthcare costs saved if he knows the expected number of participants to be enrolled in CDSMP in next 12 months (*n* = 1,000) and their anticipated age distribution. The six-step process taken by Mr. Smith utilizing the Excel-based Tool is described below. Mr. Smith entered relevant numbers marked in diagonal stripe based on his data and projections for his target region/service area (Table 4). He consulted his records and the recollection of his colleagues and partners to gather data including number of CDSMP participants (at baseline, 6 and 12 months), ER visits, hospitalizations, baseline age distribution, estimated program delivery cost, and the expected number of participants (with their anticipated new age distribution) to be enrolled in CDSMP in next 12 months.

Step 1: Examine the pattern of ER visits and hospitalizations among CDSMP participants (*n* = 500) in the first and second 6-month periods
  Table 4 shows changes in ER visits from baseline (15%) to 6 months (12%) to 12 months (11%). ER visits were reduced between baseline to 6 months (3%) and baseline to 12 months (4%). Table 4 also shows changes in hospitalizations from baseline (16%) to 6 months (11%) to 12 months (15%). Hospitalizations were reduced between baseline and 6 months (5%) and baseline to 12 months (1%).
Step 2: Identify mean costs for ER visits and hospitalizations from 2010 medical expenditure panel survey (MEPS)
  Table 4 shows mean costs for ER visits and hospitalizations from 2010 MEPS accounting for the age distribution of the large city as noted above. Then Mr. Smith identified mean costs of ER visits by the aforementioned age categories from the 2010 MEPS dataset and found $1,501.24 as the age-adjusted cost of ER visits. Thus, total cost savings associated with ER visits per person at two time periods amounted to $105.09 [first 6 months (3% reduction × $1,501.24) + second 6 months (4% reduction × $1,501.24)]. Using the same MEPS dataset, he identified $13,941.12 as an age-adjusted cost of hospitalizations, and total cost savings associated with hospitalizations per person at two time periods amounted to $836.47 [first 6 months (5% reduction × $13,941.12) + second 6 months (1% × $13,941.12)].
Step 3: Estimate costs saved from reduced ER visits and hospitalizations for two 6-month periods of CDSMP
  Table 4 shows that $941.55 was the potential annual healthcare savings per CDSMP participants from averting ER visits ($105.09) and hospitalizations ($836.47).
Step 4: Estimate average annual CDSMP costs
  Table 4 shows that Mr. Smith suggests $250 as the estimated program delivery cost per person based on the average number of participants in each workshop and the organizational capacity of providing CDSMP.
Step 5: Deduct the average annual CDSMP costs (#4) from the estimated cost savings from reduced ER visits and hospitalizations (#3)
  The potential annual net healthcare cost savings of $691.55 per participant was found by deducting the annual per participant program costs ($250) from the estimated annual per participant healthcare savings ($941.55) (Table 4).
Step 6: Project healthcare cost savings based on the expected number of participants to be enrolled in next 12 months and their anticipated age distribution.

**Table 4:** **Healthcare cost savings estimating tool: a metropolitan city**.

Healthcare cost savings estimator tool: CDSMP^a^						

National Study Case Example				Your Local Example: If you have data, please enter relevant numbers in cells marked in diagonal stripe for your population. This spreadsheet will make automatic calculations for you		

	*N*	%	Change in %	*N*	%	Change in %
**1. EXAMINE THE PATTERN OF HEALTH CARE UTILIZATION WITHIN YOUR POPULATION**						
**Emergency room (ER) visits**						
Include number of participants at baseline	1170			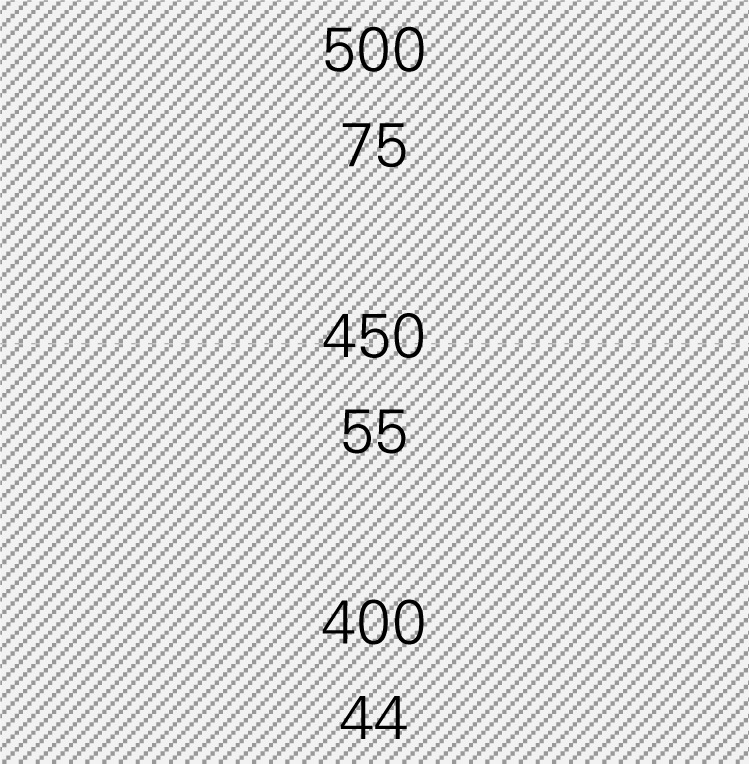		
Include number of participants reported visiting ER at baseline	211	18%			15%	
Include number of participants at 6 months	903					
Include number of participants reported visiting ER at the first 6 months	118	13%	5%		12%	3%
Include number of participants at 12 months	825					
Include number of participants reported visiting ER at the second 6 months	108	13%	5%		11%	4%
**Hospitalizations**						
Include number of participants reporting hospitalization at baseline	164	14%		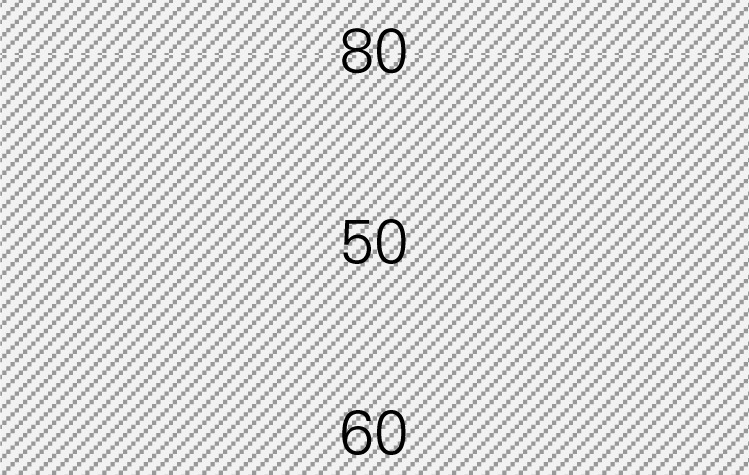	16%	
Include number of participants reporting hospitalization at the first 6 months	100	11%	3%		11%	5%
Include number of participants reporting hospitalization at the second 6 months	116	14%	0%		15%	1%
**2. IDENTIFY MEAN COSTS FOR HEALTH CARE UTILIZATION FROM 2010 MEDICAL EXPENDITURE PANEL SURVEY (MEPS)**						
**Age distribution**				**Indicate the age distribution for your population**		
Include % for those 18-44 years of age	10%			Indicate % for those 18-44	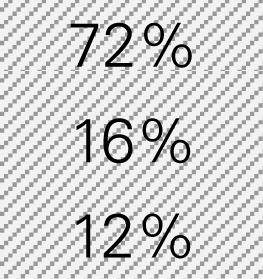	
Include % for those 45-64 years of age	31%			Indicate % for those 45-64		
Include % for those 65+ years of age	59%			Indicate % for those 65+		
**ER Visits**						
Mean costs of ER visits for those 18-44 years of age	$ 1,465.00			$ 1,465.00		
Mean costs of ER visits for those 45-64 years of age	$ 1,738.00			$ 1,738.00		
Mean costs of ER visits for those 65+ years of age	$ 1,403.00			$ 1,403.00		
Age-adjusted cost of ER visits	$ 1,513.05			$ 1,501.24		
Cost savings associated with ER visits per person at the first 6 months	$ 75.65			$ 45.04		
Cost savings associated with ER visits per person at the second 6 months	$ 75.65			$ 60.05		
Total cost savings associated with ER visits per person at two time periods	$ 151.31			$ 105.09		
**Hospitalizations**						
Mean costs of hospitalizations for those 18-44 years of age	$ 11,501.00			$ 11,501.00		
Mean costs of hospitalizations for those 45-64 years of age	$ 21,462.00			$ 21,462.00		
Mean costs of hospitalizations for those 65+ years of age	$ 18,554.00			$ 18,554.00		
Age-adjusted cost of hospitalizations	$ 18,750.18			$ 13,941.12		
Cost savings associated with hospitalizations per person at the first 6 months	$ 562.51			$ 697.06		
Cost savings associated with hospitalizations per person at the 2nd 6 months	$ –			$ 139.41		
Total cost savings associated with hospitalizations per person at two time periods	$ 562.51			$ 836.47		
**3. ESTIMATE COSTS SAVED FROM REDUCED UTILIZATION FOR THE PERIOD OF TIME YOU ARE INTERESTED IN EXAMINING**						
Based on national information, potential annual health care savings per CDSMP participant from averting ER visits ($ 151.31) and hospitalizations ($ 562.51) can be estimated	$ 713.81			Potential annual health care savings ($ 105.09 + $ 836.47)	$ 941.55	
**4. ESTIMATE AVERAGE ANNUAL PROGRAM DELIVERY COSTS**						
Estimated program delivery costs per person in the National CDSMP study	$ 350.00			Select your closest program cost per person from the drop-down menu	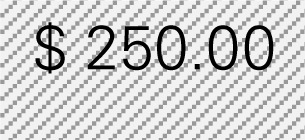	
**5. DEDUCT ANNUAL PROGRAM COSTS FROM ESTIMATED HEALTH CARE UTILIZATION SAVINGS**						
Based on national information and using average CDSMP costs per participant ($ 350.00), net cost savings related to ER visits and hospitalizations per CDSMP participant can be estimated	$ 363.81			Net cost savings ($ 941.55 − $ 250.00)	$ 691.55	
**6. EXTRAPOLATE TO NATIONAL SAVINGS USING CENSUS DATA COMBINED WITH MEPS DATA** **6. CALCULATE YOUR SAVINGS BASED ON POPULATION TO REACH AND NEW AGE DISTRIBUTIOIN**						
Number of American adults from Census data by age	234,564,071	100%		Number of potential participants reflecting their age distribution	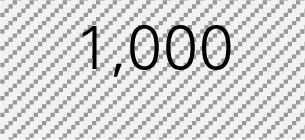	100%
18-44	112,806,642	48%		18-44	670	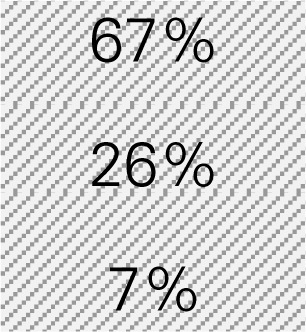
45-64	81,489,445	35%		45-64	260	
65+	40,267,984	17%		65+	70	
Estimated % of American adults having at least 1 chronic condition from MEPS data by age	77%			Net Cost Savings based on population to reach and new age distribution	732.29	
18-44	71%					
45-64	84%					
65+	94%					
Number of American adults aged 18 and older having at least 1 chronic condition	180,614,335					
Cost savings if you could reach ALL American adults age 18+ having at least 1 chronic condition	$ 65,709,373,342.03					
Include % of this population you want to reach	5%					
Based on per participant program annual net savings ($ 363.81) for the population you want to reach (5%), national health care savings can be estimated	$ 3,285,468,667.10			Your healthcare net cost savings by averting ER visits and hospitalizations attributed to CDSMP	$732,289.00	

*^a^Be aware of potential limitations when presenting your data*.

*The cost estimates presented must be treated as general estimates, as they are not based on precise cost expenditures. Yet, we feel they are robust for purposes of providing ballpark health care utilization costs, program delivery expenses, and estimated net savings to support the widespread dissemination and sustainability of evidence-based chronic disease self-management programs*.

Mr. Smith estimates $691.55 as per person net cost savings among CDSMP participants in Step 5. Now, Mr. Smith wants to project healthcare cost savings when reaching 1,000 people in the next 12 months (Table 4). He also decides to recruit 10% more middle-aged adults (i.e., from 16% to 26%) after realizing that the costs of ER visits and hospitalization for this population is more expensive than their younger or older counterparts (also shown at Table 4). As a result, when reaching more middle-aged population [i.e., 26% compared to younger (67%) and older adults (7%)], Mr. Smith estimates $732.29 as the new net cost savings, and concludes CDSMP could potentially help save $732,289 (i.e., 1,000 × $732.29). This equates to approximately $40,000 more healthcare cost savings than the age distribution of his existing CDSMP participant pool.

## Discussion

When users input the appropriate values for their situation, they will be able to use this Tool to customize estimated cost savings related to reduced healthcare utilization for participants anticipated to enroll in the CDSMP within the next 12 months. Moreover, they can then forecast net healthcare savings of an expanded recruitment or delivery effort (i.e., reaching even more participants and middle-aged or older participants in next 12 months). As illustrated in the heuristic case examples of this manuscript, the Tool can be used to estimate cost savings for CDSMP programs in different size communities. For a program planner or coordinator to customize the Tool, they will be required to supply setting-specific data that can be obtained from various sources. This may involve review of their past records as well as consultations with community and academic partners to ensure accurate information and projections are entered into the Tool. The availability of CDSMP baseline and follow-up data (including ER and hospitalization utilization) and documented per participant program costs are important for tailoring cost savings estimates. Similarly, additional tailoring is possible with access to data about the age distribution of the target community and the mean costs for ER and hospitalizations, which may be available at a county-level. Capacity to conduct such tailoring may suggest a need for technical assistance to guide program coordinators and planners to resources about how and where to locate information documenting healthcare utilizations of ER visits and hospitalizations or other necessary information for customization (e.g., age-adjusted mean costs of ER visits and hospitalizations among adults with at least one chronic condition, per person CDSMP program cost).

Healthcare savings data should be extremely useful for program administrators and key decision makers. Concrete estimates of achieved savings can bolster the impact of self-reported data on program successes. The savings estimates can also assist program administrators and decision makers in developing a strong business case to obtain funding for CDSMP and recruiting partners or sponsors from other organizations who can also benefit from reduced healthcare spending and over-utilization. The Tool will allow program coordinators to set performance goals and monitor progress in relation to the efficiency required to achieve the desired return on investment. Finally, we anticipate that users will share their results internally to their organization, externally to the community, and across geographic regions to raise public awareness about the value of CDSMP.

### Future directions

We believe developing an accessible and user-friendly web-based version of this Tool will be important for attracting a national cadre of potential users to estimate healthcare savings. When translating this Tool to a web-based interface, we plan to offer a variety of reporting features and introduce it via channels such as a national webinar, relevant health and aging services organizational websites, and social media. As a follow-up, we envision an online tutorial will be created to help different key stakeholders understand how to use (and/or collect) local or state data to estimate the amount of healthcare costs saved by CDSMP for specific populations of interest. Ideally, early users will provide feedback and suggestions to help us improve the Tool and maximize its utility. Moreover, it would be also beneficial if we could extrapolate the methodology of this Tool to create new tools to estimate healthcare cost savings associated with specific chronic conditions or participants of other evidence-based programs (e.g., enhance fitness, a matter of balance).

### Tool limitations and potential challenges

The data and methods used to develop this Tool have limitations that should be acknowledged. First, ER visits and hospitalizations were self-reported healthcare measures that could be biased. However, self-reported data can be fairly accurate for these utilization measures, as evidenced in a national study examining the concordance between self-reported and Medicare administrative data for those with Medicare claims data (Jiang et al., under review). In this prior study, we identified moderate [for ER visits; kappa statistics (0.45–0.61)] to substantial [for hospitalization; kappa (0.69–0.79)] concordance among 119 CDSMP participants (19). Next, all estimates applied in the Tool have been based on the static 2010–2012 *National Study of CDSMP*, 2010 MEPS, and 2010 Census datasets. The primary reason for this is that the national initiative to implement CDSMP started in 2010. Therefore, we synchronously utilized the 2010 MEPS and Census dataset. To keep estimates current, inflation estimators will need to be built into future iterations of the Tool. We reiterate our caution that customization of healthcare expenditures should only be attempted if there are sufficient numbers of participants with linked healthcare utilization data (we would recommend a lower threshold of at least 100 participants). The Excel-based Tool is also limited in that it generates the number of participants to reach, which is not directly linked to the target population in a given community or region. Stated another way, the Tool does not currently calculate the proportion or percentage of the population to be reached in the community based on the projected number of participants identified by the user. This may be a needed feature for public health policy makers whose “unit of analysis” could be a proportion of population to reach rather than a specific number of populations. An updated version of Excel-based Tool or Web-based Tool will reflect this feature. Additionally, the current Tool is not yet tested among users in broader fields, though plans for testing it are being currently being developed.

## Conclusion

Given findings from previous studies, CDSMP could save a significant amount of healthcare costs by averting ER visits and hospitalizations, if even only a small portion of the population was reached (15). These results are quite encouraging in that they demonstrate a positive return on investment for CDSMP nationally. The creation of this Tool contributes to the field by introducing a user-friendly resource to help program administrators and decision makers more easily estimate healthcare savings among their existing and planned CDSMP implementation efforts.

## Conflict of Interest Statement

The authors declare that the research was conducted in the absence of any commercial or financial relationships that could be construed as a potential conflict of interest.

This paper is included in the Research Topic, “Evidence-Based Programming for Older Adults.” This Research Topic received partial funding from multiple government and private organizations/agencies; however, the views, findings, and conclusions in these articles are those of the authors and do not necessarily represent the official position of these organizations/agencies. All papers published in the Research Topic received peer review from members of the Frontiers in Public Health (Public Health Education and Promotion section) panel of Review Editors. Because this Research Topic represents work closely associated with a nationwide evidence-based movement in the US, many of the authors and/or Review Editors may have worked together previously in some fashion. Review Editors were purposively selected based on their expertise with evaluation and/or evidence-based programming for older adults. Review Editors were independent of named authors on any given article published in this volume.
